# Optimising and adapting perfusion feeds in serum-free medium to intensify CAR-T cell expansion in stirred-tank bioreactors

**DOI:** 10.3389/fbioe.2025.1593895

**Published:** 2025-06-02

**Authors:** Pierre Springuel, Tiffany Hood, Fern Slingsby, Timo Schmidberger, Nicola Bevan, Noushin Dianat, Julia Hengst, Qasim A. Rafiq

**Affiliations:** ^1^ Department of Biochemical Engineering, University College London, London, United Kingdom; ^2^ Product Excellence Bioreactor Technologies, Sartorius Stedim UK Limited, Epsom, United Kingdom; ^3^ Sartorius Stedim Biotech GmbH, Goettingen, Germany; ^4^ Essen BioScience Ltd. (part of the Sartorius Group), Royston, United Kingdom; ^5^ Sartorius Stedim FMT S.A.S., Aubagne, France

**Keywords:** CAR-T cell, perfusion, xeno-and serum-free medium, adaptive, process intensification, stirred-tank bioreactor

## Abstract

The *ex vivo* expansion of autologous chimeric antigen receptor (CAR) T cells to reach a therapeutic dose significantly prolongs manufacturing time and increases overall costs. The common use of animal- or human-derived serum in T cell expansion culture media further contributes to process variability, costs and introduces additional safety concerns. To address these challenges, this study focused on intensifying CAR-T cell expansion using perfusion processes in xeno-free (XF) and serum-free (SF) culture medium. The impacts of alternative tangential flow (ATF) perfusion rates, perfusion start times and donor variability were evaluated using a Design of Experiments (DOE) approach in the Ambr^®^ 250 High-Throughput Perfusion stirred-tank bioreactor. This allowed the identification of optimal combinations of perfusion parameters on a per-donor basis, enabling 4.5-fold improvements in final cell yields and over 50% reductions in the expansion time required to reach a representative CAR-T dose compared to a fed-batch process. Subsequent process development then established an adaptive perfusion strategy enabling 130 ± 9.7-fold expansions to achieve final cell densities of 33.5 ± 3 × 10^6^ cells/mL while reducing medium requirements by 11% without compromising CAR-T cell quality attributes compared to static well-plate cultures. Harvested cells predominantly expressed naïve and central memory markers, low levels of exhaustion markers, and maintained cytotoxicity and cytokine release *in vitro*. This study demonstrates the potential of optimising and adapting perfusion strategies in XF/SF-culture medium to enhance CAR-T cell yields, shorten expansion times and reduce medium consumption while addressing patient variability in clinical manufacturing. Key considerations for future implementation and improvement of adaptive perfusion feeds for clinical CAR-T manufacturing are also discussed.

## 1 Introduction

The emergence of chimeric antigen receptor (CAR) T cell therapy has transformed haematological cancer treatment with seven FDA-approved autologous CAR-T products to date. Since the first approval in 2017 however, it is estimated that only 30,000–40,000 commercial FDA-approved CAR-T doses have been manufactured to treat patients (Levine et al., 2024). It is widely considered that complex manufacturing and logistics challenges unique to CAR-T cell therapies are still to be resolved to increase capacity of production and meet rising demand (Rafiq et al., 2019; Abou-el-Enein et al., 2021).

One such challenge is the prolonged duration of cellular expansion of patient-derived CAR-T cells that is required to reach a clinical dose (Vormittag et al., 2018). As cell quality can vary significantly between patients, often being suboptimal due to prior chemotherapy or severe disease states, cell expansion to reach a therapeutic dose represents one of the longest phases of production, typically ranging from 7–14 days (Agliardi et al., 2025; Song et al., 2024). Failure to attain a therapeutic dose due to sub-optimal cell growth has been reported as the cause of up to 13% of manufacturing failures (Jo et al., 2023; Baguet et al., 2023). Despite a recent shift in the field towards rapid or no-expansion protocols (Dias et al., 2024; Ghassemi et al., 2022), expansion steps remaining in currently approved commercial processes significantly extend overall CAR-T manufacturing turnaround time, reported to range from 16–33 days (Elsallab and Maus, 2023). Beyond escalating overall costs (∼$400K per dose), such prolonged timelines pose critical risks for patients who can face rapid cancer progression during manufacturing. Intensified expansion processes capable of generating clinical CAR-T doses in the shortest possible time are therefore required.

The continuous exchange of culture medium via perfusion is a well described method to intensify cell growth for the biopharmaceutical production of monoclonal antibodies, vaccines and viral vectors (Schaub et al., 2023). By replenishing key nutrients and removing toxic byproducts, perfusion provides a stable culture environment to promote cellular proliferation. Implementing perfusion has consequently also more recently been demonstrated as a promising strategy to enhance T and CAR-T cell growth over fed-batch processes in microfluidic, hollow-fibre and stirred-tank bioreactors (Sin et al., 2024; Smith, 2019; Coeshott et al., 2019; Nankervis et al., 2018; Hood et al., 2025). However the broad diversity of perfusion strategies described in these demonstrations highlight a lack of established perfusion strategies tailored specifically to maximising CAR-T cell yields and quality. Furthermore, unlike in stable cell line-based biopharmaceutical production, CAR-T manufacturing is faced with the additional complexity of important variability in starting cellular material which varies greatly from patient to patient. Pre-defined and fixed perfusion strategies are therefore unlikely to maximise manufacturing outcomes and address patient variability, thus requiring the need for adaptive perfusion strategies.

We previously demonstrated the successful intensification of CAR-T cell expansion using perfusion in fetal bovine serum (FBS)-containing culture medium (Hood et al., 2025). Serum continues to represent a critical raw material in current GMP CAR-T manufacturing, as the presence of growth factors, hormones, and vitamins in serum supports robust cell growth. However, the lot-to-lot variability of animal- or human-derived sera further exacerbates an already variable manufacturing process due to patient variability (Karnieli et al., 2017). The potential presence of animal or human viruses in serum also poses contamination risks, requiring extensive safety testing which further increase cost of goods. Furthermore, with the growing demand for serum driven by the rise of cell and gene therapies, estimates suggest that global serum production may be nearing its peak, raising concerns about supply limitations (Brindley et al. 2011; Gstraunthaler et al., 2013). To address these challenges, there is a consensus supported by regulatory authorities that cell therapy must transition towards xeno-free (XF) and serum-free (SF) media formulations (Karnieli et al., 2017). Accordingly, intensified perfusion processes must be coupled with XF/SF culture media to improve CAR-T process consistency while still achieving desired product quality attributes.

To this end, we employed a Design of Experiments (DOE) approach to systematically evaluate the effects of perfusion rate, initiation time, and donor variability on CAR-T cell growth, metabolism, phenotype, and *in vitro* cytotoxicity in 4Cell^®^ Nutri-T GMP, a XF/SF medium, using the Ambr^®^ 250 High Throughput Perfusion stirred-tank bioreactor. A baseline, high and adaptive perfusion strategy were then tested to assess the feasibility of further intensifying CAR-T cell expansion by reducing overall medium consumption without compromising cell yields, phenotype or *in vitro* cytotoxicity.

## 2 Results

### 2.1 Implementing and optimising perfusion parameters reduces time to first dose by 50% and increased CAR-T yields 4.5-fold

**FIGURE 1:**
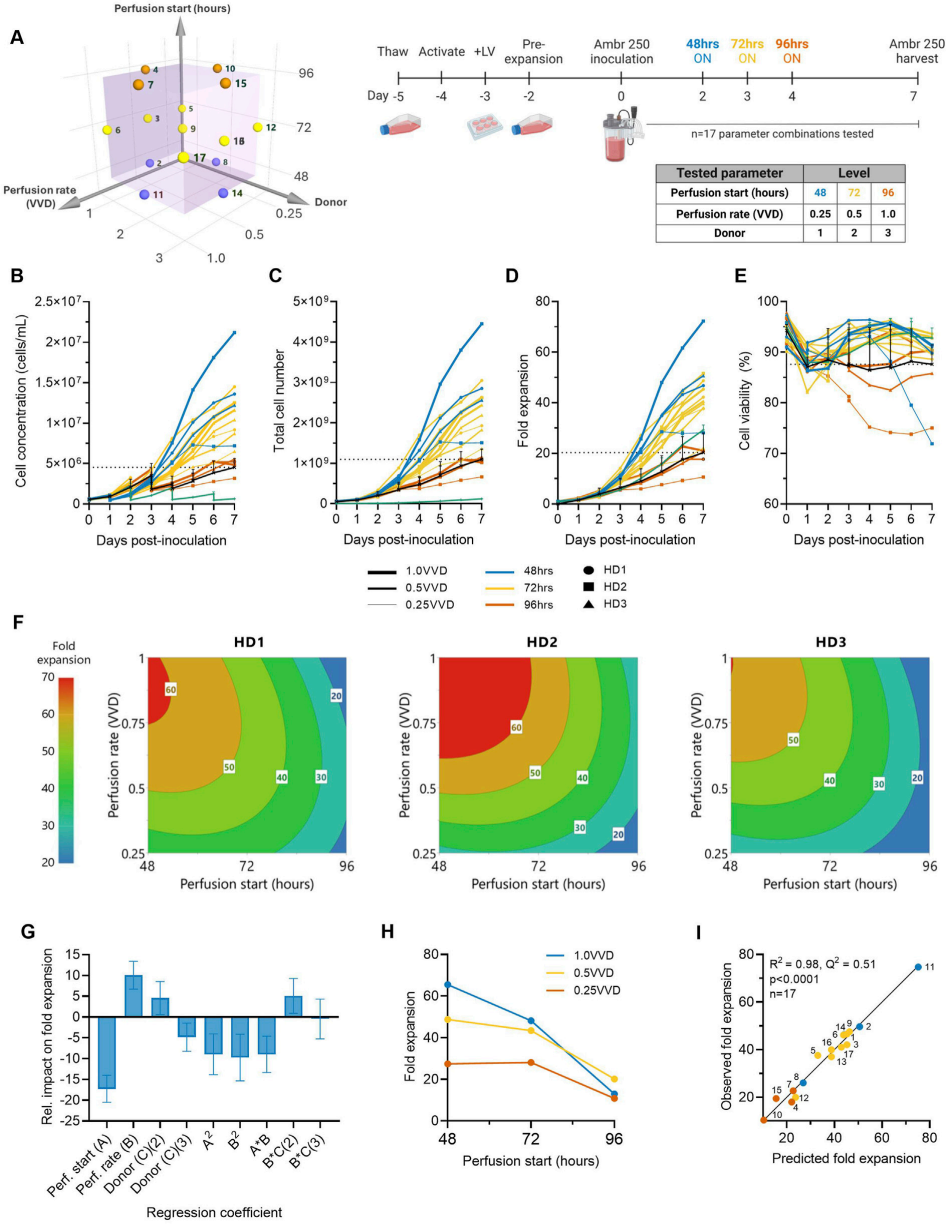
Higher ATF perfusion rates initiated earlier quadruple final CAR-T cell yields and halve the time to reach a dose. **(A)** The effects of ATF perfusion rate (0.25, 0.5, 1.0 VVD), start time (48, 72, 96 h), and donor (*n = 3*) on CAR-T cell growth in xeno-free, serum-free medium were investigated in the Ambr 250 using a half-factorial Design of Experiments (DOE) comprising *n = 17* perfusion experiments. **(B)** Daily viable cell concentration, **(C)** viability, **(D)** total cell yield, and **(E)** fold expansion. Perfusion cultures were benchmarked against an Ambr 250 fed-batch process (black line) and static T-flask cultures (green line). **(F)** The impact of perfusion rate and start time on CAR-T fold expansion was modelled using polynomial regression and visualised as contour plots for each healthy donor (HD). **(G)** Centred and normalised regression coefficients were plotted to reveal their relative contributions to fold expansion. Each regression model coefficient was assessed using ANOVA and considered significant if p < 0.05. **(H)** Interaction plot showing the combined effect of perfusion start time and rate on final fold expansion. **(I)** Correlation between model predictions and observed fold expansion. Data presented for *n = 17* Ambr 250 DOE perfusion cultures with three donors, *n = 1* fed-batch culture per donor, and *n = 3* T-flask cultures per donor. Fed-batch and T-flask data are shown as mean ± SD.

A Design of Experiments (DOE) was used to investigate the impact of perfusion initiation time (48, 72, 96 h post-inoculation), perfusion rate (0.25, 0.5, 1.0 VVD), and donor variability (n = 3) on CAR-T cell growth in XF, SF culture medium in the Ambr^®^ 250 High Throughput Perfusion stirred-tank bioreactor (Figure 1A). A total of n = 17 cultures testing different combinations of perfusion parameters and donors were performed for 7 days and benchmarked against a historical fed-batch process and static T-flask cultures.

All tested perfusion cultures were generally well tolerated and resulted in a wide range of final cell concentrations from 3.17–21.20 × 10^6^ cells/mL by Day 7 (Figure 1B). Starting with an inoculation of 50 × 10^6^ total viable cells, cells were expanded 11–72-fold, generating final cell yields ranging from 0.7–4.5 × 10^9^ total viable cells within 7 days of expansion in the Ambr 250 (Figures 1C,D). Cell viabilities typically dropped 5%–10% upon inoculation before recovering above 90% until the end of experiments in almost all perfusion cultures (Figure 1E), suggesting good CAR-T cell tolerability to the ATF-perfusion process in XF/SF medium. The continuous monitoring of the ATF cell retention filter’s transmembrane pressure showed no significant spikes in pressure, indicating no evidence of filter fouling (Supplementary Figure S1). Additionally, analysis of the permeate confirmed the absence of cells, indicating efficient retention and concentration of CAR-T cells within the bioreactor (data not shown).

Higher perfusion rates (1.0 VVD vs. 0.25, 0.5 VVD) that were initiated earlier (48 vs. 72, 96 h post-inoculation) were generally found to support the greatest CAR-T cell growth and viability (Figures 1B–D). The highest final CAR-T cell concentration of 21.20 × 10^6^ cells/mL was achieved when perfusion was initiated at the earliest tested start time (48 h) at the highest perfusion rate (1.0 VVD). In contrast, the lowest final cell concentration of 3.1 × 10^6^ cells/mL was achieved when the onset of perfusion was delayed until the latest start-time (72 h) and at the slowest rate (0.25 VVD). As expected, implementing perfusion drastically improved final cell concentrations compared to the fed-batch process in the Ambr 250 and a static T-flask control process (green and black line) (Figure 1B). The highest performing perfusion culture enabled up to 4.5-fold improvements in final cell yields compared to the fed-batch process (4.5 vs. 1 × 10^9^ cells respectively). Similarly, initiating perfusion as early as 48- or 72-h post-inoculation generally supported up to 2.5-fold greater cell fold expansions compared to the T flask process, regardless of the tested perfusion rate. Of the 17 perfusion cultures, 14 out-performed the fed-batch process both in terms of final yields and mean cell viabilities, highlighting the benefit of implementing perfusion over fed-batch, even with minimal optimisation.

In addition to increasing final cell yields, perfusion more than halved the time required to produce the equivalence of a first CAR-T dose compared to the fed-batch process (Figure 1C). Assuming a representative clinical dose of 200 million CAR + T cells, corresponding to 1 billion total viable cells at a 20% CAR transduction efficiency, the fed-batch process produced a single dose by Day 7. In contrast, the best-performing perfusion culture initiated at 48 h post-inoculation at 1.0 VVD achieved an equivalent first dose within 3–3.5 days post-inoculation and a total of 4.5 doses by Day 7, as summarised in Table 1.

**TABLE 1:** Comparison of expansion time to first CAR-T dose and total doses produced in fed-batch versus perfusion in the Ambr^®^ 250 bioreactor.

Ambr 250 process	Time to reach first dose	Total doses produced (within 7 days)
Fed-batch	7 days	1
Best DOE perfusion process	3.5 days	4.5

Assumes a representative CAR-T dose = 200 x10^6^ CAR + cells, corresponding to 1 x10^9^ total viable cells with 20% CAR+

### 2.2 Identifying donor-specific optimal perfusion parameters

The impacts of perfusion initiation time, rate and donor on CAR-T cell fold expansion were further explored using response surface analyses. Polynomial regression models were developed and visualised as contour plots to identify optimal combinations of perfusion parameter to maximise cell yields for each donor (Figure 1F). This analysis confirmed the highest fold expansions (60–70-fold) were achieved with earlier initiations of perfusion (48–60 h) and higher rates (0.75–1.0 VVD). While this optimal trend was consistent across all three donors, inter-donor variability in the optimal combinations of perfusion start time and rate was observed, as expected. This illustrated the possibility of fine-tuning perfusion parameters on donor-specific basis to account for donor variability and maximise cell fold expansion.

To further investigate the individual and combined effects of each regression model term on final cell yields, regression coefficients were centred, normalised, and plotted to reveal their relative impacts on fold expansion (Figure 1G). Perfusion start time, rate and donor all significantly impacted final cell yields, but start time emerged as the most influential factor, contributing over 50% more than perfusion rate and donor. The importance of initiation time was further emphasised in an interaction plot, which demonstrated that the effect of perfusion rate was dependent on the start time (Figure 1H). Specifically, initiating perfusion earlier amplified the benefits of higher perfusion rates on final cell yields.

The generated model for CAR-T cell fold expansion was statistically significant (p < 0.001), and accounted for most of the variability in the observed data (R^2^ = 0.975) but demonstrated more moderate predictive power (Q^2^ = 0.51), (Figure 1I). While the high R^2^ indicated a good model fit to the observed data, the moderate Q^2^ reflected the model’s limited accuracy in predicting optimal combinations of perfusion rates and start time for new unscreened donors, highlighting donor variability as an important source of uncertainty. However, as the overall trend that earlier perfusion initiation and higher perfusion rates maximised fold expansions for all three tested donors (Figure 1F), this supports the broader applicability of this strategy to maximise CAR-T cell yields generally, while more precise donor-specific parameter optimisation would require further fine-tuning. Future studies including larger and more diverse donor cohorts than those tested here would help improve model predictive power in autologous manufacturing contexts.

### 2.3 Perfusion parameters significantly impact CAR-T cell viability

**FIGURE 2:**
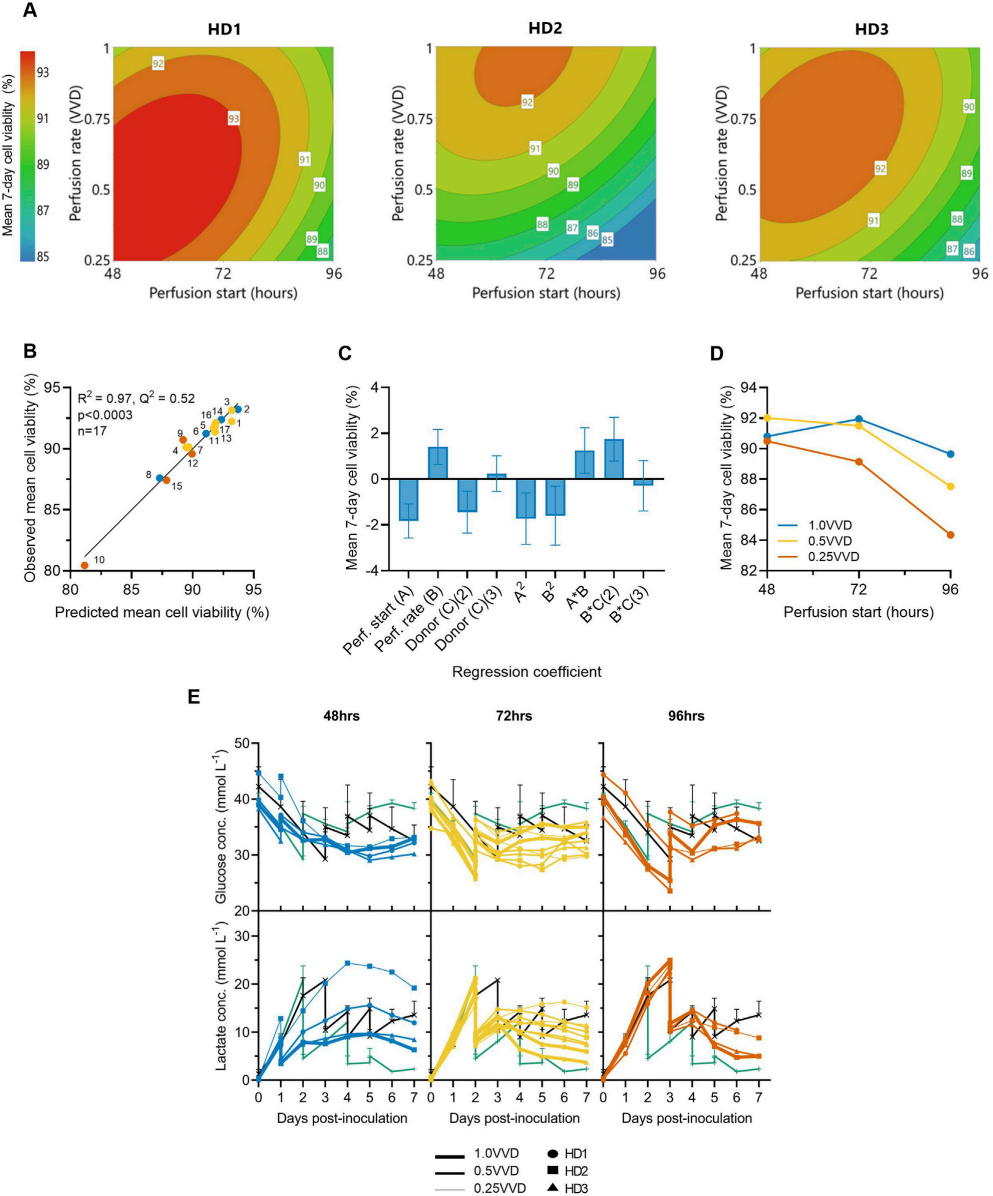
Initiating perfusion earlier and at higher rates maintains greater average cell viabilities. **(A)** The effects of ATF perfusion rate (0.25, 0.5, 1.0 VVD) and start time (48, 72, 96 h), on mean 7-day CAR-T cell viability in the Ambr 250 were modelled using polynomial regression and visualised as contour plots for each healthy donor (HD). **(B)** Correlation between model predictions and observed mean cell viability. **(C)** Centred and normalised regression coefficients were plotted to reveal their relative contributions to viability. Each regression model coefficient was assessed using ANOVA and considered significant if p < 0.05. **(D)** Interaction plot showing the combined effect of perfusion start time and rate on mean viability. **(E)** Daily glucose and lactate concentrations in Ambr 250 perfusion, fed-batch (black line), and T-flask cultures (green line). Data presented for *n = 17* Ambr 250 DOE perfusion cultures with three donor, *n = 1* fed-batch culture per donor, and *n = 3* T-flask cultures per donor (black line). Fed-batch and T-flask data are shown as mean ± SD.

Perfusion start time, rate and donor variability were each found to significantly impact mean CAR-T cell viability throughout the 7-day expansion. While minor differences in optimal perfusion parameter combination existed between donors, starting earlier and at higher perfusion rates typically maintained the highest mean viabilities >92% across all three donors (Figure 2A). The generated quadratic model was found to be significant (p < 0.001, R^2^ = 0.97) (Figure 2B), with perfusion rate, start time, donor, and squared and interaction terms each found to impact cell viability relatively evenly (Figure 2C). An interaction between perfusion rate and initiation time was observed, demonstrating the importance of operating greater perfusion rates to maintain cell viabilities the later perfusion was initiated (Figure 2D).

Perfusion cultures initiated later and at slower perfusion rates exhibited lower viabilities and were correlated with higher lactate accumulation (Figure 2E). Specifically, initiating perfusion at 48 h at 1.0 VVD consistently maintained lactate levels below 10 mmol/L, whereas initiating at 96 h at 0.25 VVD resulted in peak lactate concentrations of 24 mmol/L. Once perfusion was initiated, lactate accumulation was generally controlled and trended downward in all perfusion experiments by Day 7. This contrasted with fed-batch cultures in which lactate levels continued to rise throughout the final days of culture. Glucose depletion was never observed under any condition as elevated basal medium levels and sufficient perfusion maintained minimal glucose concentrations between 25–35 mmol/L in all perfusion experiments. Additionally, dissolved oxygen and pH levels were generally well controlled within desired set points of 50% and 7.3, respectively (Supplementary Figure S2), ensuring these parameters did not confound any effects of perfusion on cell viability.

### 2.4 CAR-T cell metabolic and growth rates decrease over time

**FIGURE 3:**
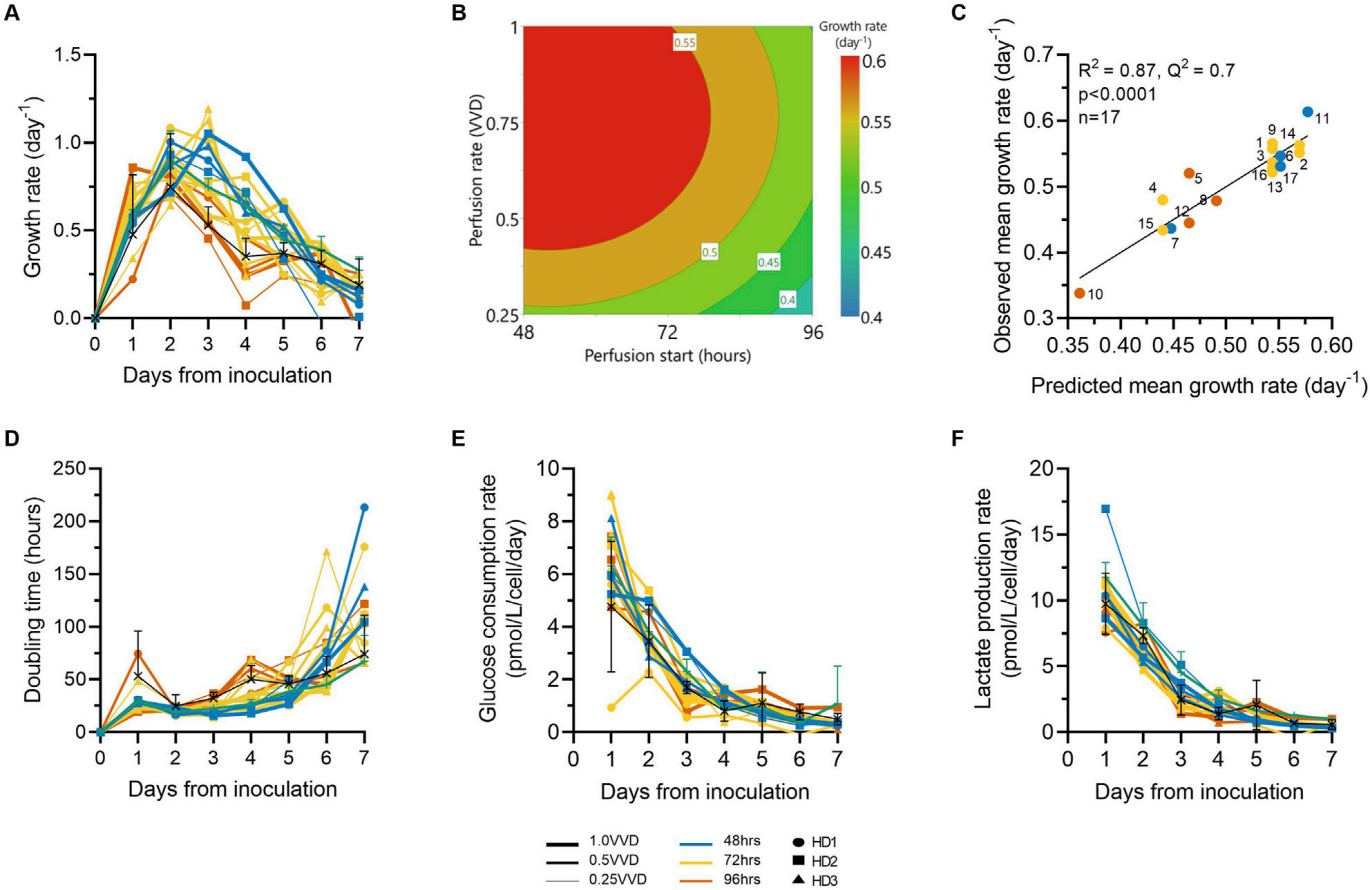
Optimising perfusion prolongs CAR-T cell growth rates, which decline over time alongside metabolic rates. The effects of ATF perfusion rate (0.25, 0.5, 1.0 VVD), start time (48, 72, 96 h), and donor (*n = 3*) in the Ambr250 perfusion experiments on **(A)** CAR-T cell growth rates. **(B)** The effects of perfusion rate and start time on average cellular growth rate across all three donors were modelled and visualised as a contour plot. **(C)** Correlation between model predictions and observed average growth rate. **(D)** Daily cell population doubling times, **(E)** glucose consumption rates, and **(F)** lactate production rates were calculated. Data presented for *n = 17* Ambr 250 DOE perfusion cultures with three donors, *n = 1* fed-batch culture per donor (black line), and *n = 3* T-flask cultures per donor (green line). Fed-batch and T-flask data are shown as mean ± SD.

CAR-T cell growth rates consistently rose then fell throughout expansion for all donors in both Ambr 250 and static flasks (Figure 3A). Post-inoculation, growth rates rose and peaked at approximately 1.0 day^-1^ by Days 2–3 before declining thereafter to approximately 0.25 day^-1^ by Day 7. The influence of perfusion parameters on mean growth rates over the 7-day culture period was effectively modelled (p < 0.001, R^2^ = 0.87), validating that earlier perfusion start times (48–72 h) and higher rates (0.5–1.0 VVD) maintained elevated growth rates for longer, irrespective of donor variability (Figure 3B, C). Cell population doubling times were correlated with growth rates, with the shortest doubling times (∼25 h) observed until Day 3 post-inoculation, later increasing to ∼75–200 h by Day 7 (Figure 3D). These dynamic changes in growth kinetics were linked with steep declines in both glucose consumption and lactate production rates immediately post-inoculation which decayed from ∼6.5 - ∼10 pmol/L/cell/day, respectively, to <1 pmol/L/cell/day by Day 7 (Figure 3E, F). The decline in cellular growth kinetics and metabolic rates were consistent for all donors and observed in all Ambr 250 perfusion and fed-batch experiments and T-flask processes.

### 2.5 Perfusion yields non-exhausted, predominantly naïve and central memory cell populations

**FIGURE 4:**
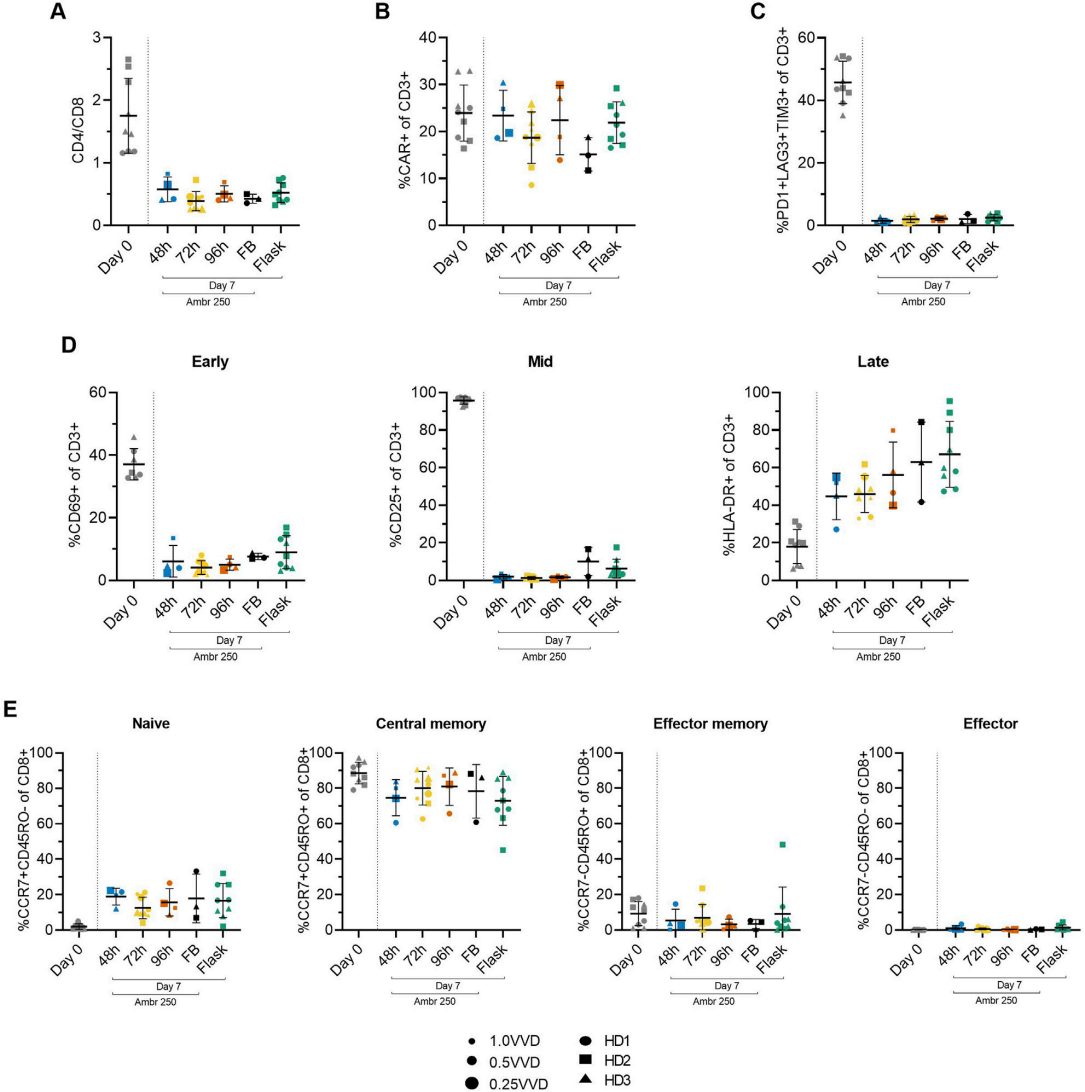
Summary of CAR-T cell surface marker expression from Ambr 250 perfusion DOE experiments, fed-batch and T-flask processes as characterised by flow cytometry at inoculation (Day 0) and harvest (Day 7). **(A)** CD4+/CD8+ ratio. **(B)** Percentage of CD3^+^ cells expressing CAR, **(C)** triple exhaustion markers, and **(D)** early, mid, and late activation markers. **(E)** Proportion of CD8^+^ cells classified as naïve (CCR7+CD45RO−), central memory (CCR7+CD45RO+), effector memory (CCR7−CD45RO+), or effector (CCR7−CD45RO−). Data presented for *n = 17* Ambr 250 DOE perfusion cultures with three donors, *n = 1* fed-batch culture per donor, and *n = 3* T-flask cultures per donor. Fed-batch and T-flask data are shown as mean ± SD, and perfusion data are shown as mean ± SD per perfusion start day.

To study the impact of the investigated perfusion parameters on cellular phenotype, surface marker expression was characterised by flow cytometry at inoculation (Day 0) and harvest (Day 7). The mean CD4/CD8+ cell ratio consistently declined from 1.7 ± 0.6 at inoculation to approximately 0.5 by Day 7 across all Ambr 250 and T-flask experiments (Figure 4A), resulting in a predominance of CD8^+^ cells at harvest. Although significant inter-donor variability was observed (Supplementary Figure S3), CAR transgene expression was generally well maintained throughout the 7-day expansion, typically ranging from 20% to 30% at harvest across all perfusion, fed-batch, and T-flask experiments (Figure 4B). Co-expression of exhaustion markers PD1, LAG3, and TIM3 decreased markedly from inoculation to harvest, falling from 46% ± 6.7% to less than 3% by Day 7 (Figure 4C). Expression of exhaustion markers at harvest remained consistently low across all experiments, including in perfusion cultures which sustained the greatest cell growth.

Activation marker expression varied overtime from inoculation to harvest, as expected (Figure 4D). Early (CD69) and mid-stage (CD25) activation marker expression of CD3^+^ cells decreased throughout the culture period from ∼37% and 96%, respectively, to <10%, while late-stage activation marker expression (HLA-DR) increased from ∼18% to 45%–67%. Interestingly, expression of HLA-DR was the most variable between perfusion, fed-batch and flask experiments. By the time of harvest, most CD8^+^ cells (>90%) were in the naïve (CCR7+CD45RO-) and central the memory subset (CCR7+CD45RO+), with the proportion of cells in central memory dropping slightly throughout the culture in favour of a rise in the naïve subset from ∼0 to 20% (Figure 4E). The proportion of effector memory (CCR7-CD45RO+) and effector (CCR7-CD45RO-) remained below 10% throughout all experiments and was not found to increase over the expansion period.

In summary, the phenotype of harvested cells was generally consistent across all tested perfusion parameters, characterised by low triple exhaustion marker expression (<5%) and a high proportion of CD8^+^ cells expressing naïve and central memory markers (>90%). The changes in activation, exhaustion, and differentiation marker expression from Day 0 to Day 7, were analysed as response variables in the DOE analyses, but these failed to reveal any significant impact of either perfusion rate or start time on measured phenotypic characteristics.

### 2.6 CAR-T cell phenotype impacted by donor and time from inoculation, not perfusion parameters

**FIGURE 5:**
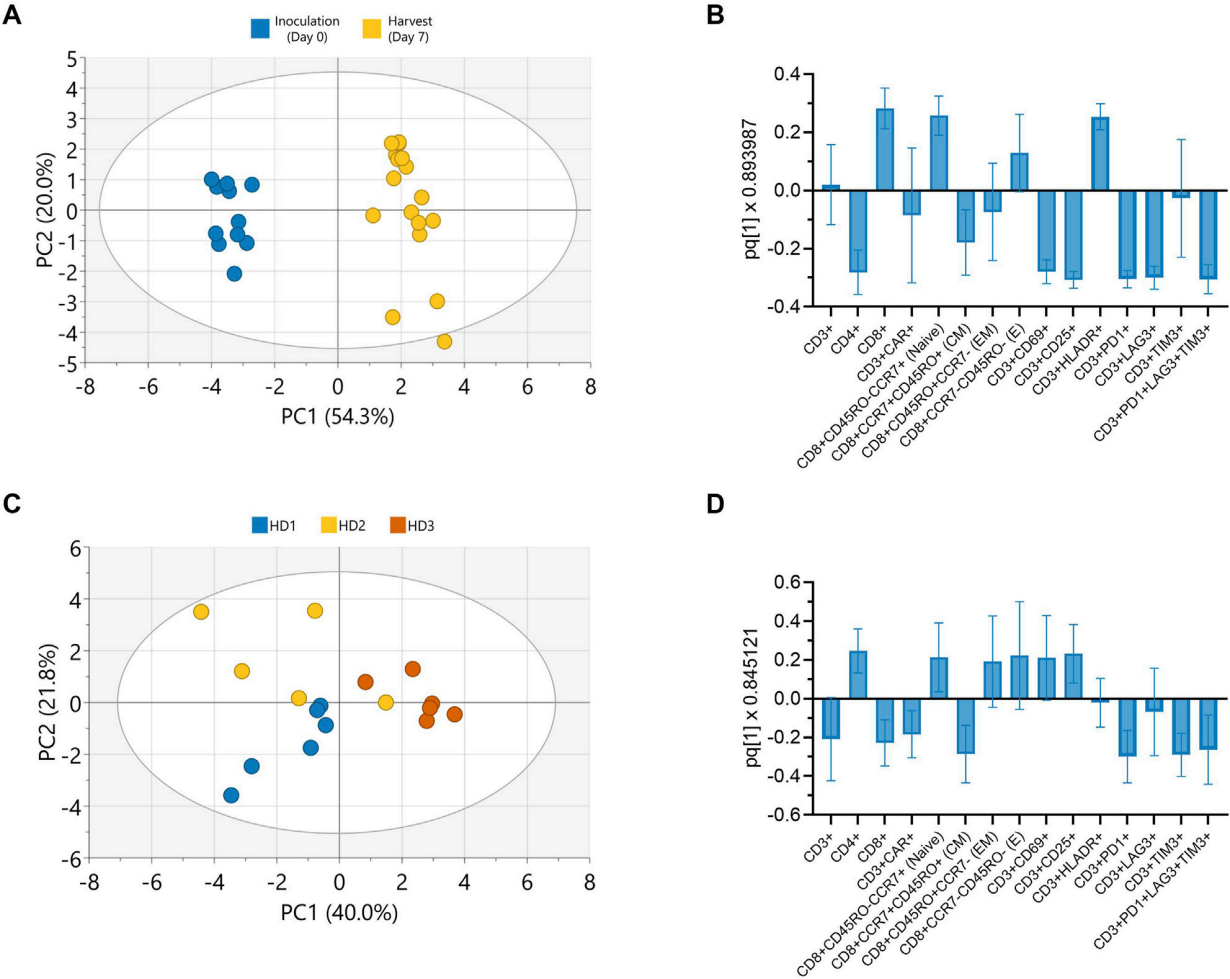
Donor and culture duration predominantly influence CAR-T cell phenotype in Ambr 250 perfusion DOE experiments. Principal component analysis (PCA) and orthogonal partial least squares discriminant analysis (OPLS-DA) were used to evaluate phenotypic variation in Ambr 250 perfusion DOE experiments. **(A)** PCA score plot illustrating the separation of phenotypic data between inoculation (Day 0) and harvest (Day 7). **(B)** OPLS-DA loadings plot identifying cell surface markers contributing to time-specific clustering. **(C)** PCA score plot illustrating donor-based separation of phenotypic data at harvest (Day 7). **(D)** OPLS-DA loadings plot identifying cell surface markers contributing to donor-specific clustering. Data presented from *n = 17* Ambr 250 DOE perfusion experiments. Score plot ellipses represent the 95% confidence interval.

Principal component analysis (PCA) was conducted on all collected phenotypic data to further explore potential patterns holistically. A score plot revealed distinct clusters between inoculation and harvest time points along principal component (PC) 1 which accounted for 54.3% of the observed variance (Figure 5A). The observed clustering suggested clear phenotypic differences between cells at inoculation (Day 0) and harvest (Day 7), consistent with trends observed in the univariate analyses (Figure 4). To further enhance cluster separation and identify the key variables contributing to these differences, an orthogonal partial least squares discriminant analysis (OPLS-DA) was performed. A single predictive OPLS component was identified, which captured the majority of variance between groups (R^2^ = 0.98) and demonstrated strong predictive performance (Q^2^ = 0.97). This reflected the high degree of separation between inoculation and harvest phenotypes. The phenotypic markers contributing most to the separation between inoculation and harvest were highlighted in the OPLS-DA loading plot, with the height and direction of each bar reflecting the strength and direction of the association, respectively (Figure 5B). Apart from CAR and effector memory marker expression, changes in most other phenotypic markers contributed significantly to the clustering observed between inoculation and harvest.

Further PCAs performed on the phenotypic data collected at harvest only, also revealed clustering based on donor (Figure 5C). The subsequent OPLS-DA loading plot highlighted that differences in CD4, CD8, CAR, naive, central memory and exhaustion marker expression mostly accounted for these donor differences (Figure 5D). Additional PCAs revealed a lack of clear data clustering based on either perfusion start time or rate, expansion platform, nor transduction, re-emphasising these parameters did not significantly impact CAR-T cell phenotype (Supplementary Figure S4). Taken together, these findings corroborate that the investigated perfusion parameters had minimal impact on CAR-T cell phenotype by harvest. Instead, donor variability and time from inoculation were the primary factors driving the observed phenotypic variation in harvested cells.

### 2.7 Adapting perfusion rates over time reduced medium consumption by 11%

**FIGURE 6:**
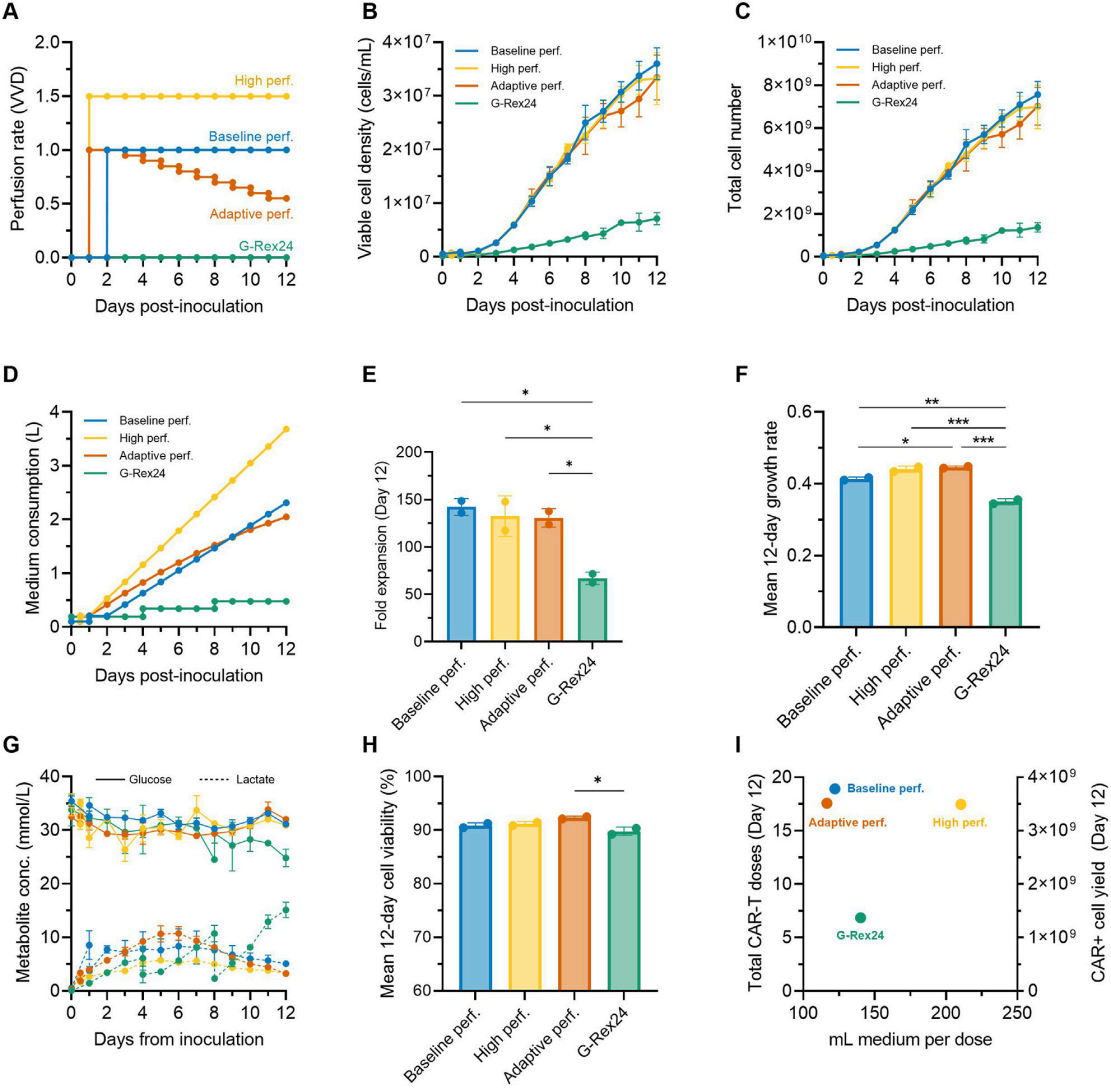
Adaptive perfusion reduces medium consumption by 11%. An adaptive 12-day CAR-T cell perfusion process in the Ambr 250 was compared to a baseline and high perfusion process and static G-Rex24 cultures. **(A)** Perfusion rate profiles for each process. **(B)** Daily viable cell concentration, **(C)** total cell yield, **(D)** total medium consumption, **(E)** final fold expansion on Day 12, and **(F)** mean 12-day cellular growth rate. **(G)** Daily glucose and lactate concentrations, and **(H)** mean 12-day viability. **(I)** Process productivity shown as total doses produced per volume of medium consumed, assuming a representative dose of 200 ×10^6^ CAR + T cells (corresponding to 400 ×10^6^ total cells assuming 50% CAR+). Theoretical total yields from G-Rex were calculated assuming 24 wells x 8 mL. Data shown as mean ± SD from *n = 2* technical replicates per condition using a single donor. Statistical significance assessed using **one-way ANOVA (*p < 0.05, **p < 0.01, ***p < 0.001).

We next explored additional strategies to further intensify the expansion of CAR-T cells in the Ambr 250 stirred-tank bioreactor while also minimising overall medium consumption. Based on our observations that CAR-T cell growth and metabolic rates declined over time (Figure 3), we hypothesised that perfusion rates could be adapted accordingly to minimise overall medium consumption. An adaptive perfusion process was therefore tested in which perfusion was initiated 24 h post-inoculation at 1.0 VVD and subsequently decreased by 0.05 VVD per day starting on Day 3 (Figure 6A). This approach was benchmarked against the optimal perfusion process from the DOE study (initiated at 48 h, 1 VVD), hereafter referred to as the baseline perfusion process. Conversely, to assess whether cell yields could be further enhanced by further increasing medium supply, a high perfusion process (initiated at 24 h, at 1.5 VVD) was also tested. Perfusion cultures in the Ambr 250 were extended from 7 to 12 days and benchmarked against gas-permeable G-Rex^®^ 24 well plate cultures using a single donor.

Cellular growth profiles were similar in all three tested perfusion processes, increasing linearly until Day 12 to reach statistically comparable final cell densities (∼33.5 × 10^6^ cells/mL) and yields (∼7.25 × 10^9^ cells) (Figure 6B, C). Compared to the baseline perfusion process, the adaptive perfusion process enabled an 11% reduction in overall medium consumption (2.05 vs. 2.30 L) (Figure 6D), while still achieving comparable final mean fold expansions (130 ± 9.7 vs. 142 ± 8.9) (Figure 6E). Interestingly, perfusing over 50% more medium (3.68 L vs. 2.30 L) in the high perfusion process, which was initiated 24 h earlier, did not result in any improvement or statistically significant difference in final fold expansions compared to the baseline process. This lack of improvement from earlier and faster perfusion rates further reinforced the optimal combination of perfusion start time and rate used in the baseline process, as identified by the DOE.

### 2.8 Perfusion in stirred-tanks supports superior CAR-T cell growth rates compared to gas-permeable well plates

Perfusion cultures in the Ambr 250 system achieved more than double the cell fold expansions over 12 days compared to G-Rex static cultures (∼135 vs. ∼67, p < 0.05) (Figure 6E). Correspondingly, average cellular growth rates were also significantly higher in perfusion experiments (p < 0.01) (Figure 6F). In G-Rex cultures, glucose depletion and lactate accumulation increased progressively until the end experiments to 24.8 mmol/L and 15.1 mmol/L, respectively (Figure 6G). This progressive lactate accumulation led to mean 12-day cell viabilities to drop just below 90% in G-Rex cultures, although this drop was only significant compared to the adaptive perfusion process (p < 0.05) (Figure 6H). In contrast, the three perfusion processes in the Ambr 250 maintained relatively stable glucose concentrations (∼32 mmol/L) and limited lactate accumulation to a maximum of 10.8 mmol/L, enabling mean cell viabilities to remain above 90% (Figure 6H). Despite notable differences in peak lactate concentrations between the baseline (5.7 mmol/L), high (8.3 mmol/L), and adaptive perfusion processes (10.8 mmol/L), no significant difference in average cell viability was observed, suggesting that lactate ranges below 10.8 mmol/L had negligible impact on cell viability.

To assess production efficiency in terms of total CAR-T doses and medium utilisation, total CAR-T doses and medium consumption per dose were plotted (Figure 6I). The number of representative clinical doses was calculated assuming of 200 million CAR + T cells, corresponding to 400 × 10^6^ total viable cells at a 50% CAR transduction efficiency. Differences in inoculation cell numbers between the G-Rex and Ambr 250 (24 × 10^6^ vs. 50 × 10^6^ cells) were adjusted by doubling the final doses generated in G-Rex cultures. Over 12 days, perfusion processes in the Ambr 250 yielded 17–19 representative CAR-T doses compared to seven doses in the G-Rex. Although the adaptive and baseline perfusion processes required five times more medium overall than G-Rex cultures (Figure 6D), these processes were approximately 15% more efficient on a per-dose basis in terms of medium efficiency, consuming around 119 mL per dose versus 140 mL per dose in the G-Rex (Figure 6I).

### 2.9 Adaptive perfusion yields predominantly naïve and central memory, functional CAR-T cells

**FIGURE 7:**
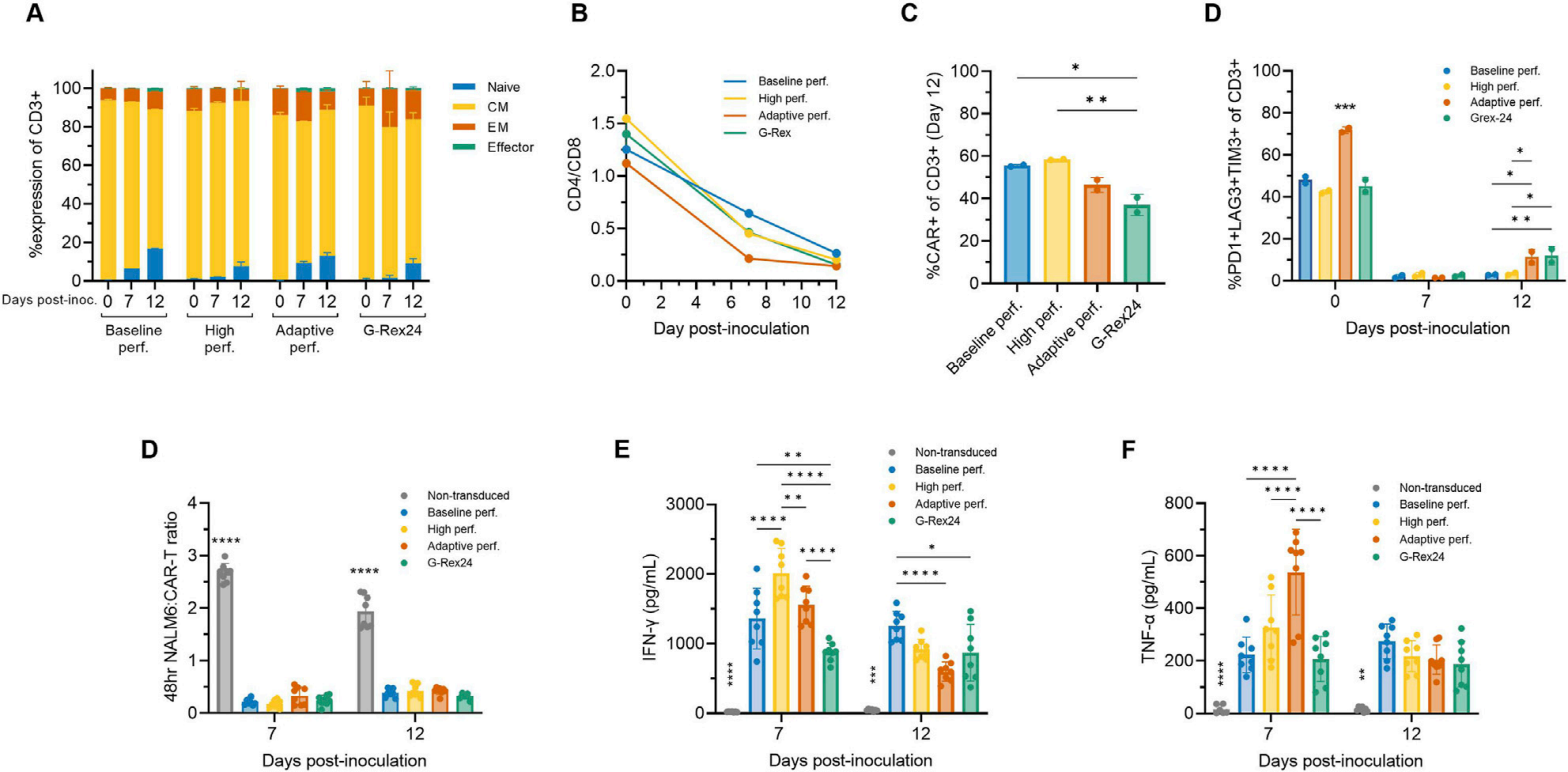
Adaptive perfusion in Ambr 250 produces cytotoxic CAR-T cells. The effects of baseline, high, and adaptive Ambr 250 perfusion processes, as well as G-Rex24 cultures, were assessed on CAR-T cell phenotype and cytotoxicity at Days 0, 7, and 12. **(A)** Proportion of CD8^+^ cells classified as naïve (CCR7+CD45RO-), central memory (CM) (CCR7+CD45RO+), effector memory (EM) (CCR7-CD45RO+), or effector (CCR7-CD45RO-). **(B)** CD4+/CD8+ ratio. **(C)** Percentage of CD3^+^ cells expressing CAR, and **(D)** exhaustion markers. Phenotypic data shown as mean ± SD from *n = 2* technical replicates per condition using a single donor. **(E)** Cytotoxicity was assessed by co-culturing purified harvested CAR-T cells with CD19^+^ NucLight Green + NALM6 target cells at a 1:1 ratio, analysed using the Incucyte^®^ S3 Live-Cell Analysis System. **(F)** Interferon-gamma (IFN-γ) and (g) tumour necrosis factor-alpha (TNF-α) concentrations were measured in cytotoxic assay supernatants collected after 48 h. Cytotoxicity data shown as mean ± SD from *n = 4* technical replicates per condition. Statistical significance was assessed using two-way ANOVA (*p < 0.05, **p < 0.01, ****p < 0.0001).

To assess the impact of adaptive and high perfusion strategies, flow cytometry was performed on Days 0, 7, and 12 across additional perfusion Ambr 250 and G-Rex experiments. By Day 12, more than 90% of the harvested CD8^+^ cells were in the naïve (CCR7+CD45RO-) or central memory (CCR7+CD45RO+) subsets (Figure 7A). The proportion of effector cells (CCR7-CD45RO-) remained relatively constant throughout the culture, even beyond Day 7 to Day 12. The previously observed shift in the CD4/CD8 ratio towards predominantly CD8^+^ populations progressed further, evolving from approximately 1.25 at inoculation to 0.5 by Day 7 and 0.25 by Day 12 (Figure 7B). Average CAR transduction efficiencies were comparable in all Ambr 250 perfusion conditions by Day 12, ranging from 46% to 58% (Figure 7C). However, mean CAR efficiencies in the G-Rex well plates were significantly lower at 33.5% compared to the baseline and high perfusion processes (Figure 7C). As observed previously, co-expression of exhaustion markers PD1, LAG3, and TIM3 on CD3^+^ cells decreased markedly from 40% - 70% at inoculation to <3% by Day 7 across all conditions (Figure 7D). Extending the culture to Day 12 did highlight a small rise in exhaustion marker expression in the adaptive perfusion and G-Rex processes, which rose to approximately 10%, significantly higher than in the baseline and high perfusion processes, which remained below 3%. Two-way ANOVA revealed that both time and process significantly impacted final exhaustion levels (p < 0.0001), with time post-inoculation accounting for 90.16% of the variation. These findings suggest that extending the expansion period by five additional days contributed to small but significant increases in exhaustion marker expression, although this trend was not observed in the baseline or high perfusion processes.

The cytotoxicity of harvested CAR-T cells was assessed *in vitro* by co-culturing purified CAR-T cells harvested on Days 7 and 12 with CD19+ NALM6 target cells in a 1:1 ratio. CAR-T cells from both Ambr 250 and G-Rex cultures effectively eliminated NALM6 cells, reducing the effector-to-target cell ratio to <0.3 within 48 h (Figure 7D). No significant differences in cytotoxicity were observed between Day 7 and Day 12, as confirmed by two-way ANOVA, indicating that neither culture time nor process significantly impacted *in vitro* cytotoxicity. Analysis of supernatant from the cytotoxicity assays confirmed that CAR-T-mediated NALM6 killing was associated with the secretion of IFN-γ and TNF-α across all conditions at both time points (Figures 7E, F). Significant differences in cytokine secretion were particularly notable on Day 7, with inter-process differences diminishing by Day 12 for reasons that remain unclear. Cytokine concentrations were also found to significantly differ over time (p < 0.001), tending to decrease from Day 7 to Day 12.

## 3 Discussion

### 3.1 Serum-free medium perfusion to intensify CAR-T cell manufacturing

Novel processes to intensify CAR-T cell expansion in SF media are essential to reduce manufacturing time, cost, and improve process consistency. Here, we demonstrate successful intensification of CAR-T cell expansion through the implementation and optimisation of ATF perfusion parameters using 4Cell^®^ Nutri-T GMP XF, SF culture medium in the Ambr 250 stirred-tank bioreactor.

Response surface analyses from our DOE optimisation study identified optimal perfusion parameter combinations to maximise CAR-T cell growth and viability on a per-donor basis. Earlier perfusion initiation combined with higher rates enabled superior cellular growth rates, yields, and viabilities, enabling up to 72-fold expansions within 7 days. The maximum cell densities of 21–36 × 10^6^ cells/mL that were ultimately achieved in this study matched or exceeded those we and others have previously reported in serum-containing perfusion processes (Hood et al., 2025; Gatla et al., 2022), confirming the suitability of new serum-free media formulations for high-density CAR-T expansion. The optimised perfusion process cut the expansion time required to reach a representative CAR-T dose by over 50% relative to a fed-batch process. These findings position perfusion as a key intensification strategy to accelerate CAR-T therapy manufacturing and increase capacity, as widely demonstrated in the production of other biopharmaceutical products (Schaub et al., 2023; Xue et al., 2024; Klimpel et al., 2025).

Reducing expansion time through perfusion optimisation could enhance CAR-T products efficacy, as faster manufacturing is increasingly linked with better therapeutic outcomes *in vivo* (Agliardi et al., 2025; Ghassemi et al., 2021). The study’s insights on perfusion parameter optimisation could be readily applied to bioreactors with filter-based perfusion capabilities currently used in commercial CAR-T manufacturing, such as the Biostat^®^ RM and Xuri™ rocking bioreactors (Hupfeld et al., 2020; Smith, 2020). For other commonly used commercial platforms without filter-based perfusion, such as the CliniMACS Prodigy^®^ or Cocoon^®^ Platform, automated batch medium additions, exchanges or recirculation could help replicate the benefits of perfusion in enhancing CAR-T cell expansion to shorten expansion times to reach a therapeutic dose (Song et al., 2024; Trainor et al., 2023).

Beyond CAR-T therapies, perfusion is well suited to intensify the production of other T cell-based therapies requiring greater doses sizes such as tumour-infiltrating lymphocytes (TILs). The dose size for the recently approved autologous TIL therapy for advanced melanoma, *lifileucel,* can require up to 72 billion total viable cells (Parums, 2024) which corresponds to 300 times more cells than standard CAR-T doses (Rotte et al., 2022). This has necessitated extended *ex vivo* expansion, with median TIL manufacturing durations reported as long as 34 days (Onimus et al., 2021). As shown in this study with CAR-T cells, perfusion processes have also been reported to significantly increase TIL yields (Sadeghi et al., 2011; Albrecht et al., 2023), offering a promising solution to shorten manufacturing times while also meeting high-dose requirements.

Besides achieving rapid cell expansion and high yields, it is essential to preserve the phenotype and cytotoxic functionality of CAR-T cells which correlate with clinical efficacy (Monfrini et al., 2022). Across our experiments, the majority of cells harvested from the Ambr 250 were primarily in naïve and central memory subsets and expressed negligible levels of exhaustion markers expressions, characteristics that are associated with improved persistence *in vivo*. Implementing perfusion to in XF/SF culture medium did not compromise *in vitro* cytotoxicity and enabled high cell densities without negatively impacting CAR-T cell quality attributes. Furthermore, this study addressed existing concerns regarding T-cell shear sensitivity as CAR-T cells were successfully cultured despite the use of perfusion and impeller-based agitation which impart higher shear stresses relative to static cultures (Carswell and Papoutsakis, 2000). The maintenance of cell viability above 90% in the absence of serum, a known shear-protectant (Kunas and Papoutsakis, 1990), not only challenges the perceived shear sensitivity of T cells and the need for static, undisturbed monolayers for optimal growth, but also underscores the robustness of the SF medium to culture high-viability CAR-T cells, even in an agitated culture environment.

### 3.2 Adapting perfusion to reduce medium consumption

As cost of goods such as culture media represent major cost drivers in CAR-T manufacturing, it is critical to minimise consumption of medium in a CAR-T perfusion process (Vormittag et al., 2018). Post-activation, CAR-T cells exhibit a dynamic proliferation pattern with an initial lag phase, rapid expansion, and subsequent slowing of growth. This offers an opportunity to adapt perfusion rates over time to match changing cellular metabolic demands, potentially reducing medium consumption by avoiding excessive perfusion of culture medium during later and slower growth phases.

We hypothesised that gradually decreasing perfusion rates could sustain CAR-T cell growth while reducing overall medium usage and characterised an adaptive perfusion strategy involving the gradual decrease of perfusion rates by 0.05 VVD per day. This approach successfully enabled a 11% reduction in overall medium consumption while maintaining comparable CAR-T cell yields, phenotype, and cytotoxicity as a baseline process with a fixed perfusion rate. Despite continuously rising cell densities, the gradually decreasing perfusion rates still effectively prevented glucose depletion and lactate accumulation due to the progressively declining glucose consumption and lactate production rates observed during the expansion phase. These metabolic trends aligned closely with those previously reported in healthy donor-derived CAR-T cells in various bioreactor systems (Sin et al., 2024; Coeshott et al., 2019). Separately, we characterised a high perfusion strategy involving fixed rates of 1.5 VVD, which consumed 50% more medium overall but did not result in any improvements in cell yields. This underscored the presence of a “perfusion ceiling,” beyond which additional perfusion failed to further enhance CAR-T cell yields and highlighted the importance of adapting perfusion rates over time to prevent excessive medium consumption.

Overall medium consumption must be considered in relation to the total number of CAR-T doses produced, reflecting the productivity of a CAR-T perfusion expansion process. While the Ambr 250 perfusion process required approximately five times more medium compared to the G-Rex system, 120 mL of medium were required per generated dose compared to 140 mL per dose in the G-Rex. This highlights the value of perfusion in significantly intensifying the productivity of CAR-T manufacturing processes, a benefit that has also been demonstrated in the production of other biopharmaceutical products (Schaub et al., 2023).

A key feature of the perfusion process described in this work was the inclusion of a pre-expansion step in flasks prior to bioreactor inoculation. As a result, cells inoculated into the bioreactor had already reached near-peak growth rates. Inoculating the bioreactor immediately post-transduction would have consequently likely required higher initial perfusion rates (>1.0 VVD) to support rapid growth and prevent lactate accumulation, as demonstrated in previous studies which implemented superior perfusion rates than those tested in this work during this early CAR-T cell growth phase (Song et al., 2024; Sin et al., 2024; Coeshott et al., 2019; Janas et al., 2015). A fully optimised adaptive perfusion process initiated immediately post-transduction would therefore likely include elevating initial perfusion rates to support early growth, followed by a gradual reduction to align with gradually decreasing CAR-T cell dynamics. Providing a perfusion strategy was adjusted accordingly, inoculating cells earlier in the Ambr 250 bioreactor without a flask pre-expansion, may have enabled final cell concentrations greater than the maximum ∼36 × 10^6^ cells/mL we achieved within 12 days.

### 3.3 Adapting perfusion in serum-free media to address variability in CAR-T manufacturing

A major challenge to overcome in autologous CAR-T manufacturing is the variability in cellular material caused by differences in patient biology and disease states (Vormittag et al., 2018). A fixed perfusion expansion process therefore cannot be expected to consistently achieve optimal outcomes when starting cellular material varies widely. Whilst our DOE response surface models indicated that earlier initiation of perfusion at higher rates generally maximised yields irrespective of donor, significant donor-specific differences in the optimal combination of perfusion parameters were still observed. These highlight the potential to fine-tune perfusion initiation times and rates on a donor-basis to account for donor variability and further improve yields and viability in autologous manufacturing.

In addition to donor-specific adaptation, the adaptation of a perfusion process can also be considered temporally, whereby perfusion rates are adjusted over time throughout an expansion process to align with changing CAR-T cell metabolic requirements. Combining both temporal and donor-specific adaptations of perfusion feeds could enable therapy developers to manage biological variability and increase process consistency, maximise desired manufacturing outcomes such as yields while also reducing mediumconumption by avoiding over-or under-perfusion. While further work is required, adapting perfusion rates to meet cellular demands may also improve CAR-T cell phenotype, as progressively increasing perfusion rates was recently reported to generate a higher proportion of naïve and central memory CAR-T cells compared to a fixed perfusion rate process (Sin et al., 2024).

The batch-to-batch variability of animal- or human-derived serum commonly included in CAR-T cell culture medium further aggravates the patient-driven variability in CAR-T manufacturing processes. In addition to increasing process consistency, the elimination of animal-derived products also eliminates safety concerns around viral contamination risks and the additional costs associated with safety testing of serum. The results in this study which were achieved exclusively in XF/SF medium add to the growing body of evidence supporting the feasibility of culture CAR-T cells to high cell densities while maintaining favourable phenotypic and functional characteristics (Coeshott et al., 2019; Eberhardt et al., 2023). These findings reinforce the viability of serum-free media for functional CAR-T manufacturing and support the ongoing shift away from serum-containing media, as recommended by regulatory agencies (Karnieli et al., 2017).

### 3.4 Considerations for adaptive CAR-T perfusion control strategies

Moving towards adaptive CAR-T perfusion processes raises key questions about optimal perfusion control strategies and upon which parameters these should be based upon. The literature presents a range of control strategies optimised for monoclonal antibody and vaccine production (Nikolay et al., 2020; Maria et al., 2023). One approach, used in this study, is vessel-volume-per-day (VVD)-based control, in which perfusion rates are adjusted based on total bioreactor volume, independent of cell concentration. While effective for more predictable stable cell lines, VVD-based strategies may be inadequate to fine-tune perfusion to address patient variability in CAR-T cell expansion, risking over- or under-perfusion. A second approach, cell-specific perfusion rate (CSPR)-based control, commonly used in monoclonal antibody production, adjusts rates according to cell concentration, ensuring a consistent provision of medium volume per cell (Maria et al., 2023; Wolf et al., 2019). However, in CAR-T manufacturing which aims to maximise cell densities without cell bleeds, a fixed CSPR-based strategy could require increasingly larger perfusion volumes, becoming cost-prohibitive. Lastly, metabolite-based perfusion control involves adjusting rates to maintain optimal nutrient levels for cell proliferation by preventing depletion of glucose and glutamine and accumulation of toxic by-products lactate and ammonia. This approach could be particularly well aligned to match dynamic and patient-specific CAR-T cell growth rates and metabolic needs to optimise yields and quality attributes.

Metabolite-based control of perfusion for CAR-T cell expansion requires an understanding of metabolic conditions that are optimal for CAR-T cell proliferation and quality more widely. In this study, as glucose concentrations remained above 20 mmol/L, glucose depletion was not a limiting factor. Instead, lactate accumulation emerged as a critical factor. Our DOE study showed that initiating higher perfusion rates earlier promoted greater CAR-T cell expansion and reduced lactate build-up. Cell viabilities were found to drop below 90% when lactate levels exceeded approximately 15 mmol/L and persisted for more than 48 h, indicating a toxic lactate threshold. This threshold aligns with previous studies showing adverse effects on the viability and proliferation of healthy human and murine T cells *in vitro* at comparable lactate levels (Tu et al., 2021; Quinn et al., 2020; Uhl et al., 2020; Fischer et al., 2007). Interestingly, in T cell perfusion processes in different bioreactors, authors focused medium feeding strategies to prevent lactate accumulation beyond 15–20 mmol/L (Coeshott et al., 2019; Janas et al., 2015). We therefore propose minimising lactate below a threshold of 10–15 mmol/L as a key parameter for metabolic-based adaptive perfusion control in CAR-T cell expansion, alongside preventing glucose depletion. As this study only included healthy donor-derived CAR-T cells, further investigations would be required to confirm whether patient-derived CAR-T cells exhibit similar lactate sensitivity. Separately, amino acid analyses which were not performed in this study may also inform on potential targeted supplementation strategies to mitigate proliferation limitations beyond glucose and lactate dynamics.

Accurate control of adaptive CAR-T perfusion processes will ultimately require real-time monitoring of biomass and key metabolites such as glucose and lactate. This can be achieved by integrating advanced process analytical tools (PATs) to enable on-line monitoring of these parameters. Emerging GMP bioreactors for CAR-T manufacturing are starting to include integrated sensors for tracking of glucose and lactate concentrations (Nettleton et al., 2023). Integrating these measurements into control algorithms could automate perfusion adjustments in real-time, ensuring that critical metabolic parameters stay within target thresholds for optimal CAR-T cell expansion (Wan et al., 2024; Esmonde-White et al., 2022; Baradez et al., 2020). Alternatively, for existing GMP CAR-T manufacturing paltforms in which implementing additional PATs may prove impractical, leveraging sensors already existing in these platforms such as those for dissolved oxygen and pH, could also provide an indirect control method. Mechanistic modelling of CAR-T cell biomass and metabolic rates derived from dissolved oxygen data has recently been demonstrated, for example, (Sin et al., 2024). Automating perfusion rate control in real-time, as is commonly done for pH and dissolved oxygen, could improve process consistency and enhance CAR-T manufacturing outcomes on per-patient basis.

### 3.5 Conclusion

Taken together, this work demonstrates that CAR-T cell expansion can successfully be intensified in XF/SF medium using perfusion and confirm that both perfusion start time and rate are critical parameters that require optimisation to significantly increase CAR-T cell growth and viability. We highlight the potential of optimising perfusion processes on a donor-to-donor basis to address biological variability and of also adapting perfusion rates to meet the changing metabolic requirements of expanding CAR-T cells over time to minimise medium consumption. Metabolic-based control of adaptive aimed at minimising lactate accumulation specifically is proposed as a potential strategy to maximise CAR-T cell growth and viability. These findings may help reduce the *ex vivo* expansion time to reach a dose in current autologous CAR-T and TIL therapy manufacturing and contribute towards maximising the total doses produced per batch to produce future universal allogeneic CAR-T modalities.

## 4 Methods

### 4.1 Primary T-cell isolation

Primary human T cells were isolated using a pan T cell isolation kit (Miltenyi Biotec, United Kingdom) from healthy donor peripheral blood mononuclear cell material obtained from leukopaks (BioIVT, United Kingdom). Post-isolation, cells were cryopreserved in Cryostore CS10 (Sigma-Aldrich, United Kingdom) at 50 × 10^6^ cells/mL and stored in liquid nitrogen until experimentation began.

### 4.2 Lentiviral vector production and titration

A second-generation lentiviral vector encoding an anti-CD19 CAR (shared by Dr Martin Pule, University College London) was produced in-house via plasmid transfection of adherent HEK293T cells. Adherent HEK293T cells were transfected and cultured in 10STACK flasks (Corning, United Kingdom) for 48 h in DMEM medium (Thermo Fisher Scientific, United Kingdom). Vector-containing medium was harvested, filtered (0.45 μm) and stored at −80°C. Vector was later thawed and titrated using in a JURKAT-based transduction assay and flow cytometry. The CAR transgene contained an epitope recognisable by CD34^+^ antibodies, allowing for determination of transduction efficiency via flow cytometry and magnetic purification of CAR + T cells.

### 4.3 CAR-T cell production

Isolated T-cells were thawed and rested overnight at 2 × 10^6^ cells/mL. Cells were activated the following day via addition of CD3/CD28 Dynabeads (Thermo Fisher Scientific, United Kingdom) at 1 × 10^6^ cells/mL in a 1:1 bead:cell ratio, supplemented with 30 IU/mL IL-2 (Miltenyi Biotec, United Kingdom). One-day post-activation, cells were transduced with lentiviral vector at an MOI = 3 in Retronectin-coated well-plates (20 ug/cm^2^) (Takara, France), supplemented with IL-2 and spinnoculated at 1000 g for 40 min. Cells and virus were co-incubated overnight, after which CAR-T cells were resuspended in fresh culture medium. CAR-T cells were seeded in T-flasks at 0.5 × 10^6^ cells/mL, supplemented with IL-2 and pre-expanded for 2 days before inoculation of different bioreactors.

All experiments were entirely performed in 4Cell^®^ Nutri-T GMP Medium PRF (Sartorius, United Kingdom), a xeno-free, serum-free cell culture medium, supplemented with 1% (v/v) Antibiotic-Antimycotic (Thermo Fisher Scientific, United Kingdom).

### 4.4 Ambr 250 stirred-tank bioreactor culture

One day prior to bioreactor inoculation, custom un-baffled Ambr^®^ 250 vessels were installed into the Ambr^®^ 250 High Throughput perfusion bioreactor (Sartorius, United Kingdom) and filled with medium. The following day, 50 × 10^6^ cells total cells were inoculated in the bioreactor in a total working volume of 100 mL, supplemented with 30 IU/mL IL-2. The bioreactor working volume was increased to 210 mL 1 day prior to the onset of perfusion (ranging from 24-, 48-, 72- and 96-h post-inoculation), and maintained at that volume thereafter. ATF perfusion via a 0.2 μm hollow-fibre filter was operated at fixed rates of either 0.25, 0.50, 1.00 or 1.50 volume vessel per day (VVD) as specified. For the adaptive perfusion process, perfusion was started 24 h post-inoculation and maintained at 1 VVD for 2 days, after which perfusion rate was decreased by 0.05 VVD per day until the end of culture. For the high perfusion process, perfusion was started at 24 h post-inoculation and fixed at 1.5 VVD. Fresh bottles of medium and new permeate bags were replaced throughout experiments as required.

For fed-batch cultures, the culture working volume was increased from 100 mL to 200 mL on Day 3, and to 250 mL on Day 4 post-inoculation via automated liquid addition. On day 5, a 40% medium exchange was performed by manually removing 100 mL of cell culture and resuspending and returning cells in fresh medium. Throughout all Ambr 250 experiments, batch and perfusion medium feeds were always supplemented with 30 IU/mL IL-2 (Miltenyi Biotec, United Kingdom).

The bioreactor elephant-ear impellor agitation rate was set at 200 RPM and temperature controlled at 37°C. Dissolved oxygen (DO) levels were allowed to drop naturally to 50% and controlled at that level thereafter via headspace gassing of oxygen. pH was controlled at 7.35 using headspace gassing of carbon dioxide and addition of 1 M sodium carbonate base solution (Sigma-Aldrich, United Kingdom).

### 4.5 G-Rex24 and T-flask culture

Each G-Rex^®^ 24 well plate (Wilson Wolf Manufacturing, United States) was inoculated on Day 0 with 1 × 10^6^ total cells in 8 mL working volume per well supplemented with 30 IU/mL IL-2. Every 2 days, IL-2 was supplemented at the same concentration and a 75% medium exchange was performed in each well every 4 days. Daily cell counts and metabolite analyses were performed using sacrificial well sampling.

T-flasks (Sarstedt, Germany) were inoculated with cells at 0.5 × 10^6^ cells/mL and supplemented with 30 IU/mL IL-2. Cultures were diluted back down to 0.5 × 10^6^ cells/mL and supplemented with IL-2 every 2 days thereafter. Excess cells were discarded as required to maintain the cell culture within a T-75 flask.

### 4.6 Cell counting

Cell samples were taken from all expansion platforms immediately post-inoculation and daily thereafter for cell count and viability analysis using the NucleoCounter^®^ NC-3000™ cytometer (ChemoMetec, Denmark).

### 4.7 Flow cytometry

Flow cytometry was performed on fresh CAR-T cells on the day of bioreactor inoculation and harvest using L/D for viability staining. Expression of T-cell subset markers CD3, CD4, CD8 (BD Biosciences, United Kingdom), differentiation markers CCR7, CD45RO (BD Biosciences,United Kingdom), CAR expression via CD34 (R&D Systems, United Kingdom) were analysed on the LSRFortessa™ X-20 flow cytometer (BD Biosciences, United Kingdom). Data analysis was performed in FlowJo™ v10. Activation (CD25, HLA-DR, CD69) and exhaustion (PD1, LAG3, TIM3) marker expression was determined using the respective iQue^®^ kits and the iQue^®^3 High-Throughput Screening (HTS) cytometer (Sartorius, United Kingdom), according to the manufacturer’s instructions.

### 4.8 Calculation of representative CAR-T doses

The total number of representative doses was calculated by multiplying the total number of viable cells at harvest by the CAR transduction efficiency and assuming 200 × 10^6^ total CAR + cells per dose. In the DOE perfusion optimisation experiments the average CAR transduction for all three donor at harvest was used in the calculation. In the subsequent adaptive perfusion experiments the average CAR transduction efficiency of the single additional donor across all experiments was used in the calculation.

### 4.9 Metabolite analysis

Cell samples were taken daily from all expansion platforms, centrifuged and the supernatant frozen at −80°C until analysis. Samples were later thawed and analysed for glucose and lactate concentrations using the CuBiAn HT270 analyser (4BioCell, Germany).

### 4.10 CAR-T cytotoxicity assay and cytokine analysis

On the day of bioreactor harvests, CAR-T cells were purified via magnetic separation using CD34 isolation kit and LS Columns (Miltenyi Biotec, United Kingdom) according to the manufacturer’s instructions. Purified CAR-T cells were rested overnight in T-flasks at 2 × 10^6^ cells/mL. The following day, 15,000 CAR-T cells were co-cultured in 96-well plates 1:1 with target CD19+, Nuclight Green+ NALM6 cells and analysed using the Incucyte^®^ S3 Live-Cell Analysis System (Sartorius, United Kingdom). Four replicate 20X microscopy images were taken per well, every 2 hours throughout-out the 2-day co-culture.

At the end of the 2-day co-culture medium supernatant samples were taken from each cytotoxicity assay well and frozen at-80°C until analysis. Medium samples were later thawed and levels of tumour necrosis factor (TNF) alpha and interferon (IFN) gamma were analysed using the iQue^®^3 HTS cytometer (Sartorius, United Kingdom).

### 4.11 Design of experiments and statistical analyses

A half-factorial Design of Experiments was performed to investigate the impact of perfusion start time (48, 72, 96 h), perfusion rate (0.25, 0.50, 1.00 VVD) and healthy donor (Levine et al., 2024; Rafiq et al., 2019; Abou-el-Enein et al., 2021) on CAR-T cell growth and phenotype. A total of n = 17, 7-day perfusion cultures were performed in the Ambr 250 testing different combinations of the perfusion parameters, as summarised in Supplementary Table S1. Measured responses were analysed, modelled and visualised using MODDE v12 (Sartorius, United Kingdom).

Principal component analysis (PCA) and orthogonal partial least squares discriminant analyses (OPLS-DA) were performed in SIMCA v18 (Sartorius, United Kingdom). Data figures were generated and analysed in GraphPad Prism 9 (GraphPad, United States), using t-tests, one-way and two-way ANOVAs as required. Overview figures were created using BioRender (BioRender, Canada).

## Data Availability

The raw data supporting the conclusions of this article will be made available by the authors, without undue reservation.
