# Supplementary material for: Optimising and adapting perfusion feeds in serum-free medium to intensify CAR-T cell expansion in stirred-tank bioreactors
Source: Front Bioeng Biotechnol. 2025 Jun 2;13:1593895. doi: 10.3389/fbioe.2025.1593895 (PMC12171161; doi:10.3389/fbioe.2025.1593895)
Supplement: Supplementary file 1 [file DataSheet1.docx]

Supplementary Material


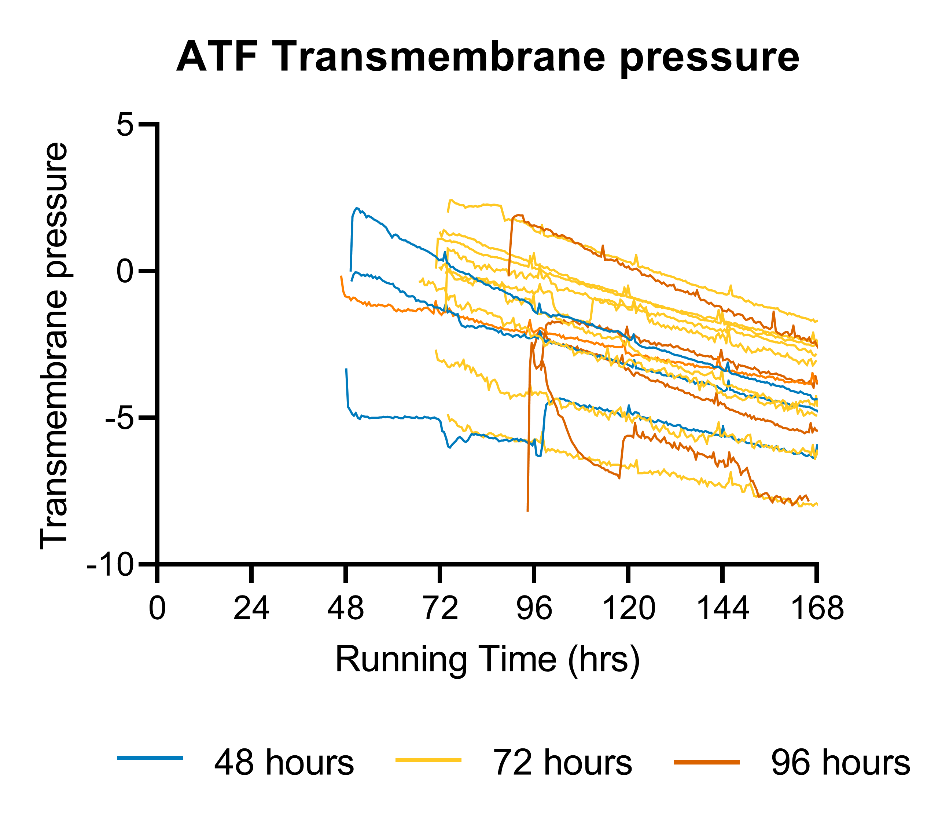


**Supplemental Figure 1: On-line alternating tangential flow (ATF) perfusion filter transmembrane pressures in the Ambr250.** Trends represent the ATF transmembrane pressures from n=17 perfusion experiments were performed testing the impact of perfusion rates (0.25, 0.5, 1.0VVD), perfusion start time (48, 72 ,96 hours) and donor (n=3) on CAR-T cell expansion.


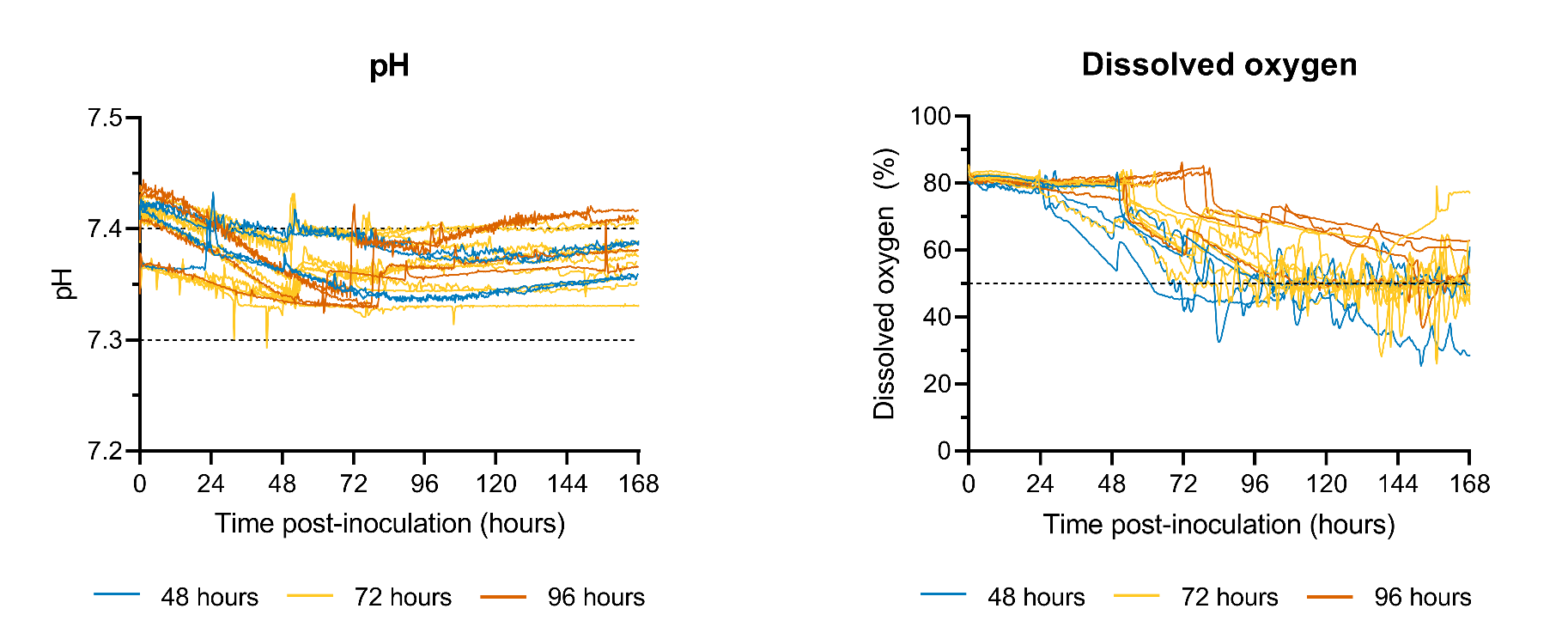


**Supplemental Figure 2: On-line pH and trends dissolved oxygen in the Design of Experiments (DOE) perfusion cultures in the Ambr250.** A total of n=17 cultures were performed testing the impact of perfusion rates (0.25, 0.5, 1.0VVD), perfusion start time (48, 72 ,96 hours) and donor (n=3) on CAR-T cell expansion.


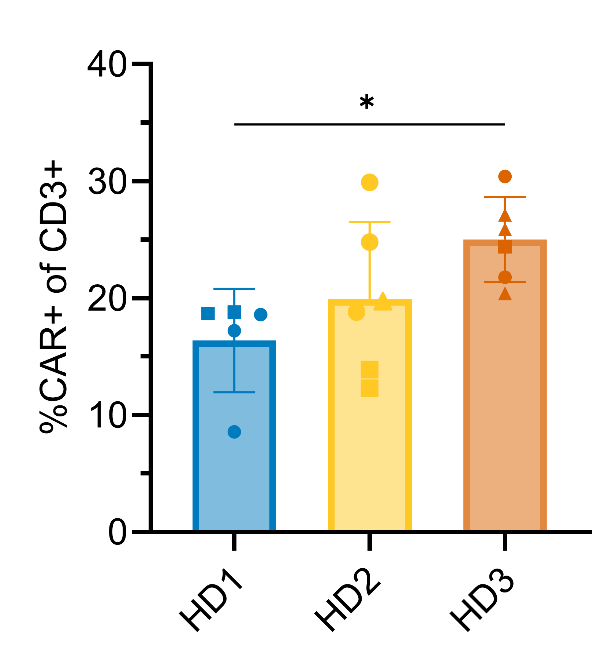


**Supplemental Figure 3: Percentage of harvested cells expressing CAR transgene in the n=17 Ambr250 perfusion cultures (Day 7)**. Data shown as mean ± SD. Statistical significance measured via one-way ANOVA. *p<0.05. HD=Healthy donor


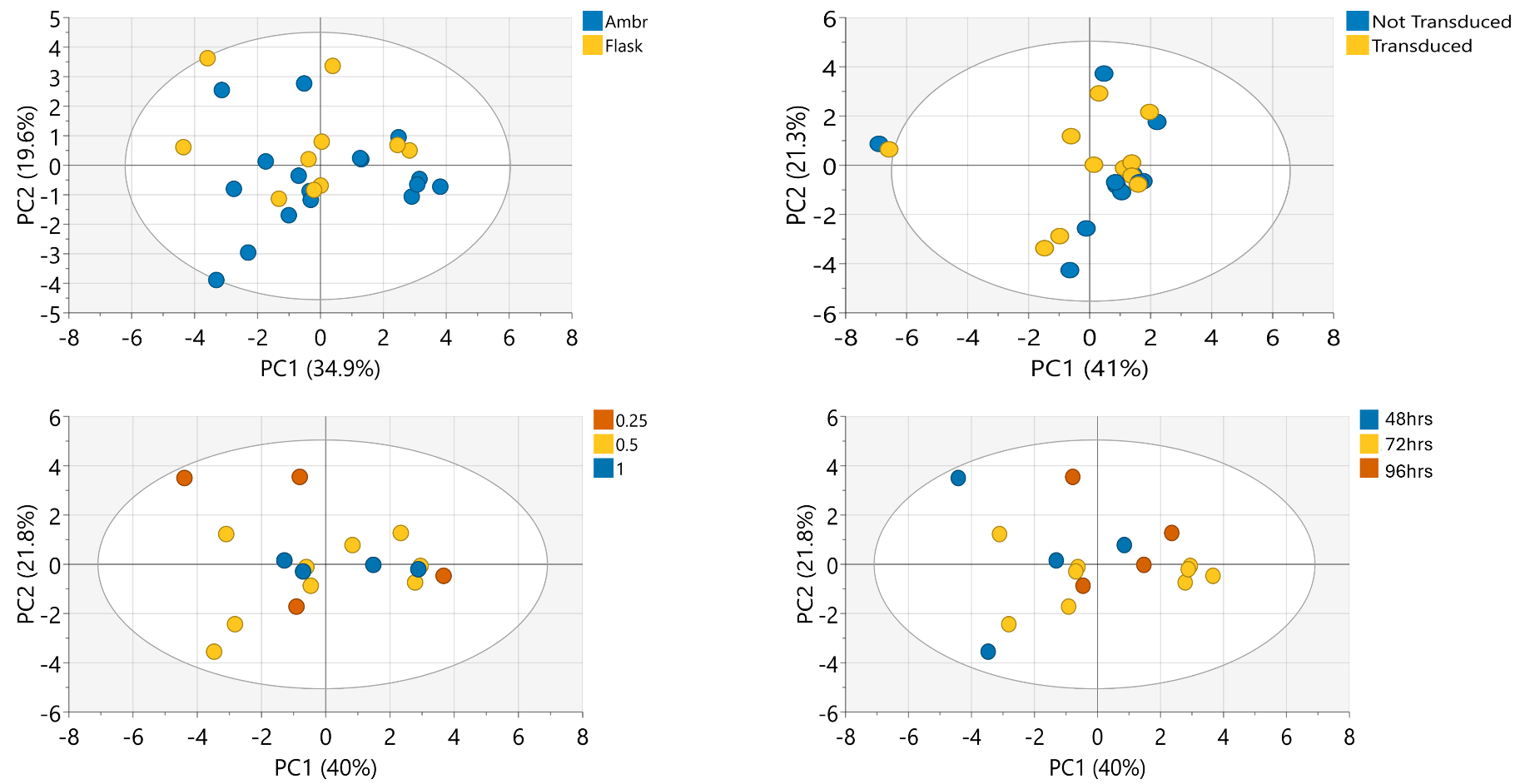


**Supplemental Figure 4: Principal component analyses (PCA) of perfusion Design of Experiments study in the Ambr250 reveals lack of data clustering based on expansion platform, transduction, perfusion rate or perfusion start time. a)** PCA score plot illustrating unclear separation of phenotypic data based on Ambr250 versus T-flask process. **b)** PCA score plot illustrating unclear separation of phenotypic data between transduced and non-transduced flask controls. PCA score plots illustrating unclear clustering based on **c)** perfusion rate (0.25, 0.5, 1.0VVD and **d)** perfusion start time (48. 72 .96 hours post-inoculation). Ellipses represent the 95% confidence intervals for each group.

**Supplemental Table 1:** Overview of tested parameter combinations of perfusion start time, perfusion rate and donor investigated in the n=17 Design of Experiment perfusion runs in the Ambr250 and

| **DOE Run** | **Feed start day** | **Perfusion rate (VVD)** | **Donor** |
| --- | --- | --- | --- |
| 1 | 2 | 0.5 | Donor 1 |
| 2 | 1 | 0.5 | Donor 1 |
| 3 | 2 | 0.5 | Donor 1 |
| 4 | 3 | 0.5 | Donor 1 |
| 5 | 2 | 0.25 | Donor 1 |
| 6 | 2 | 1 | Donor 1 |
| 7 | 3 | 1 | Donor 2 |
| 8 | 1 | 0.25 | Donor 2 |
| 9 | 2 | 0.5 | Donor 2 |
| 10 | 3 | 0.25 | Donor 2 |
| 11 | 1 | 1 | Donor 2 |
| 12 | 2 | 0.25 | Donor 3 |
| 13 | 2 | 0.5 | Donor 3 |
| 14 | 1 | 0.5 | Donor 3 |
| 15 | 3 | 0.5 | Donor 3 |
| 16 | 2 | 0.5 | Donor 3 |
| 17 | 2 | 1 | Donor 3 |
